# Group refractive index via auto-differentiation and neural networks

**DOI:** 10.1038/s41598-023-29952-8

**Published:** 2023-03-17

**Authors:** G. Alagappan, C. E. Png

**Affiliations:** Fusionopolis, Institute of High-Performance Computing, Agency for Science, Technology, and Research (A-STAR), 1 Fusionopolis Way, #16-16 Connexis, Singapore, 138632 Singapore

**Keywords:** Integrated optics, Optics and photonics

## Abstract

In this article, using principles of automatic differentiation, we demonstrate a generic deep learning representation of group refractive index for photonic channel waveguides. It enables evaluation of group refractive indices in a split of second, without any traditional numerical calculations. Traditionally, the group refractive index is calculated by a repetition of the optical mode calculations via a parametric wavelength sweep of finite difference (or element) calculations. To the direct contrary, in this work, we show that the group refractive index can be quasi-instantaneously obtained from the auto-gradients of the neural networks that models the effective refractive index. We embed the wavelength dependence of the effective index in the deep learning model by applying the scaling property of the Maxwell’s equations and this eliminates the problems caused by the curse of dimensionality. This work portrays a very clear illustration on how physics-based derived optical quantities can be calculated instantly from the underlying deep learning models of the parent quantities using automatic differentiation.

## Introduction

Interest in fundamental and applied research in photonics is steadily growing in recent years. Therefore, accelerated design and modelling tools are vital for disruptive improvement in the state-of-the-art photonic components and systems. The photonic parameter space is finite and well defined. For instance, we know the operating wavelengths, the exact material(s) composition, and the range for the fabrication feasible geometrical dimensions. Researchers often explore this limited parameter space repeatedly using brute-force numerical methods whenever they initiate a new design task. Given the perspectives of the current machine learning era, such repeated explorations would utilize compute resources inefficiently. Any finite parameter space can be captured, and an effective representation can be formulated. Such representation often assumes deep learning models and have been proven to be a solid replacement for the traditional photonic models^1–7^. Sophisticated techniques like automatic differentiation (AD)^8–13^ can be further applied to these models, to evaluate derived quantities without any further training or computational effort. To the best our knowledge, this is the first investigation of derived quantities in photonics using neural networks and AD. Here, we used AD to evaluate group refractive index—a derived quantity of paramount importance in photonic design and discovery. Group refractive index is derived from the parent quantity, effective refractive index.

The field of AD, having a history of few decades is not new. However, the recent pull of interest towards machine learning techniques drives creation of sophisticated AD libraries such as PyTorch^14^, Tensorflow^15^, Autograd^16^ and Jax^17^. These libraries are widely used in many areas of computational science and engineering. For example, AD is a key enabler in solving differential equations using neural networks^18–24^. In photonics, AD has been successfully applied in inverse design problems designing L3 photonic crystal cavities^25^ and multifunctional meta-surfaces^26^.

In this work, we illustrate the effortless computation of group refractive index for the entire and usual parameter space of photonics [generic in wavelength, materials, and geometrical parameters]. We employ deep learning for surrogate modelling of effective refractive indices and use AD to arrive at a sophisticated framework for quasi-instantaneous group refractive index computation. Group refractive index plays a significant role in photonics. The quantity is key to perceive the wavelength response of any given photonic geometry. It is a crucial parameter in computing the free spectral range of an optical cavity and estimating photon lifetimes and laser thresholds. In order to compute the group refractive index, traditionally one performs wavelength sweeps of optical mode calculations^27,28^. This sweep is done numerically using the time and memory expensive matrix diagonalization techniques. To the direct contrary, here, we show that as a consequence of AD, the value of group index emerges naturally from the weights of the deep learning models that predicts the effective refractive index. In other words, if one has constructed the deep learning model for effective refractive index, the group index is a natural side product which can be effortlessly obtained without any further training or laborious calculations.

**Figure 1:**
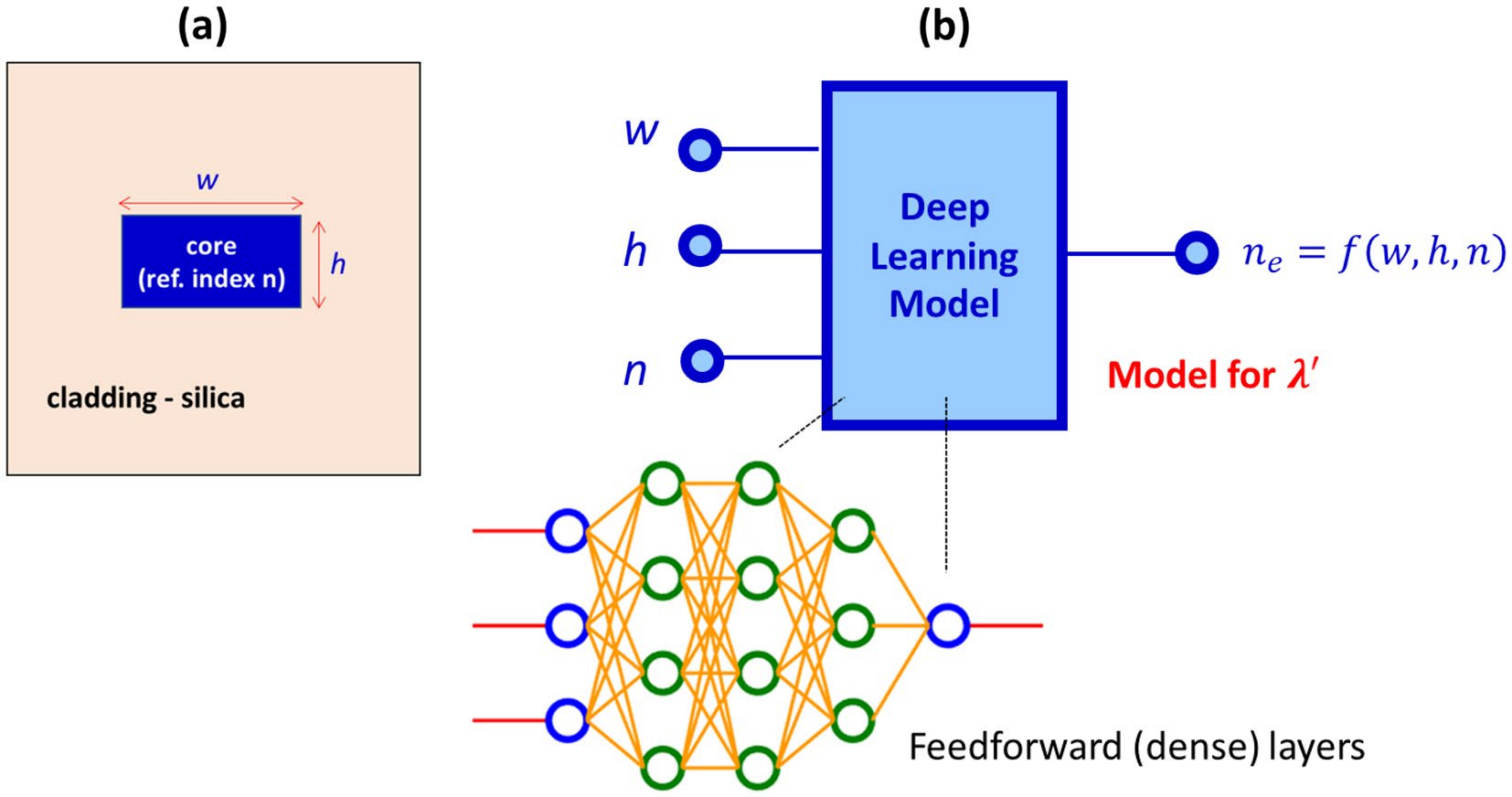
(**a**) Schematic of the channel waveguide. (**b**) The deep learning model for the effective refractive index.

For the sake of an illustration, we considered an optical channel waveguide with a rectangular geometry and light with TE polarization. The waveguide has width and height of *w* and *h* respectively (Fig. 1a). We consider core and cladding materials as dielectric materials. For most applications in photonics, cladding material can be conveniently fixed to be silica with refractive index of 1.45, while the core refractive index (*n*), *w*, and *h* varies in the ranges of 1.45 to 4, 0.2 to 1.0 μm, and 0.2 to 0.5 μm, respectively. These geometrical dimensions are well within the typical photonic fabrication capabilities. For waveguide applications, one of the key requirements on the cladding and core materials is the optical transparency. Therefore, we assumed the material loss is considerably small, and do not take the imaginary part of the refractive index into account. This will also allow us to be consistent with the earlier works^29^ on the deep learning prediction of effective refractive index, where materials with real refractive index values were assumed.

Figure 1b shows the schematic of the deep learning model for an effective refractive index prediction. The input to the model is the vector consisting of *w*, *h,* and *n* and the output is the effective refractive index $\left({n}_{eff}\right)$. The deep learning model approximates the function $f$ where ${n}_{eff}=f\left(w,h,n\right)$. The model depicted in Fig. 1, has been trained thoroughly earlier^29^ for wavelength, λ′ = 1.55 micron. In particular, the optimizations of the feedforward architecture (i.e., number of layers and number of neurons) has been rigorously carried out for varying data set sizes.

Group refractive index is defined as,

1 $$n_{g} = n_{eff} - \lambda \frac{{dn_{eff} }}{d\lambda }$$

Group refractive index is the direct consequence of the wavelength response of the effective refractive index. So, in the first place, we should include wavelength ($\lambda$) in the deep learning model. How can wavelength be incorporated in the deep learning model? One simple way is to increase the input vector size in Fig. 1a by including the wavelength as an additional component and retrain the model. However, increasing the dimension of the input vector makes requires a much bigger data set to train (curse of dimensionality). Additionally, wavelengths can continuously vary and hence the training can be tremendously laborious. A more computationally elegant way is to apply the scaling property of Maxwell’s equations. There is no fundamental length scale in Maxwell’s equations. This allows devices to be modelled at different wavelengths by simply scaling the geometrical dimensions in proportion to the change in wavelengths.

**Figure 2:**
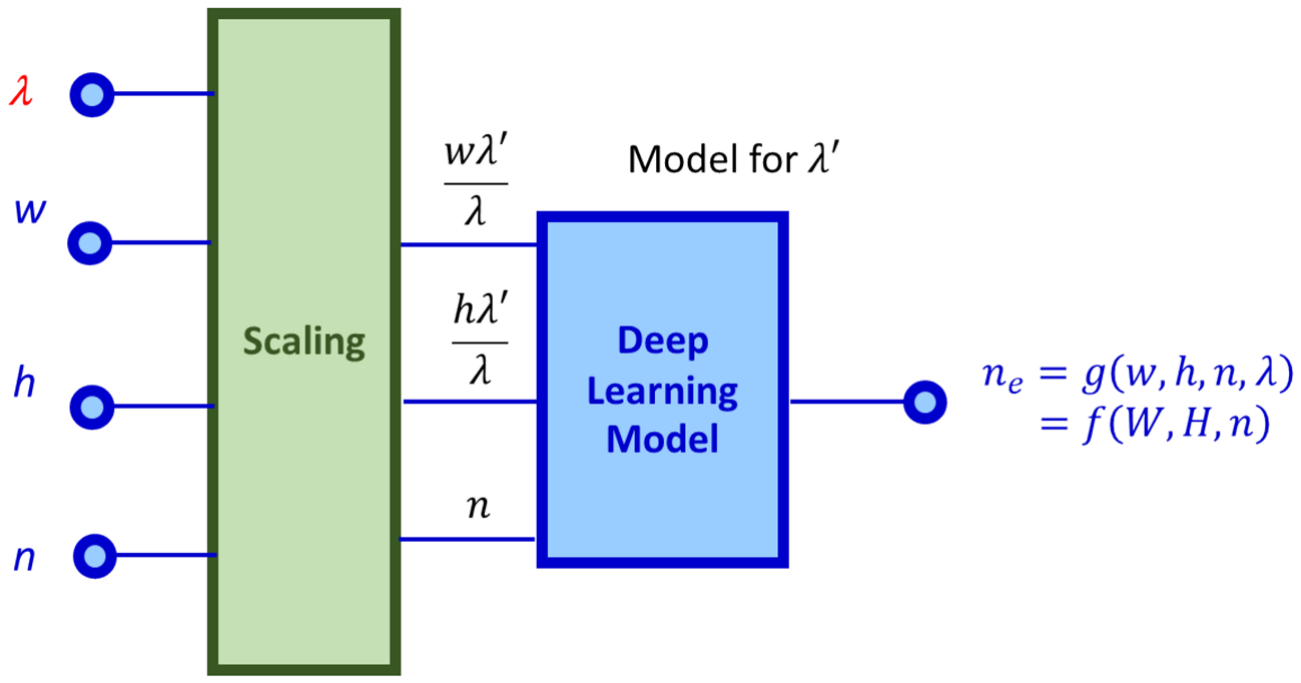
The block diagram of the wavelength generic deep learning model.

Figure 2 shows the wavelength generic deep learning model. The model uses the architecture for λ′ = 1.55 µm [color coded in blue], with a scaling layer [color coded in green] upfront. Note that in this model, the training is required only for λ′. Once the model is trained for λ′, we can use the same model (i.e., same architecture and weights) to predict the effective refractive indices for other wavelengths by simply scaling the input geometrical parameters.

Let us write the output of the model in Fig. 2 as ${n}_{eff}=g\left(w,h,n,\lambda \right)$. With the scaled geometrical parameters, $W=w\lambda^{\prime}/\lambda$ and $H = h\lambda^{\prime}/\lambda$, the effective refractive index can be written as $n_{eff} = g\left( {w,h,n,\lambda } \right) = f\left( {W,H,n} \right)$. Subsequently, the total differential becomes $dn_{eff} = {\frac{\partial f}{{\partial W}}dW + \frac{\partial f}{{\partial H}}}dH + \frac{\partial f}{{\partial n}}dn$. Neglecting chromatic dispersion and with repeated applications of chain rule it can be shown that $- \lambda \frac{{dn_{eff} }}{d\lambda } = w\frac{df}{{dw}} + h\frac{df}{{dh}}$. Here the derivatives $\frac{df}{dw}$ and $\frac{df}{dh}$ can be readily evaluated using AD as ${f}_{w}$ and ${f}_{h}$, respectively. With the auto-gradients ${f}_{w}$ and ${f}_{h}$, the group is index in (1) can be re-written in the context of the deep learning model as

2 $$n_{g} = n_{eff} + wf_{w} + hf_{h}$$

This is remarkable as now the group index can be calculated without any further training or numerical evaluations. The auto-gradients ${f}_{w}$ and ${f}_{h}$ are function weights of the deep learning model used to predict the effective refractive index. The auto-gradient functions are readily available in all popular AD libraries mentioned earlier, and they automatically evaluate the gradients by simply multiplying the weight of the internal layers as per the underlying chain rule-based graphs. This computation is done in a split of a second. Therefore, if one constructed the deep learning model for effective refractive index, the group index is a natural side product which can be calculated quasi-instantaneously.

For effective refractive index modelling, we adapted the architecture developed in Ref.^29^. In Ref.^29^, a rigorous architecture optimization was carried out for photonic data sets of varying size. Table 1 shows the optimized neural network architecture as a function of *N*_*g*_. Here, *N*_*g*_ represents number of data points sampled for each input parameter. For instance, the data set with *N*_*g*_ = 5 consists of 5^3^ data points with 5 variations in each of the input quantity (i.e., *w*, *h* and *n*). The data points within each data set are further split as training, and validation according to a ratio of 0.7 and 0.3, respectively. The validation data points are not used in training, but they are used to monitor the validation loss which signals overfitting of the deep learning model. The models were trained using Levenberg–Marquardt (LM) backpropagation algorithm^30,31^.

**Table 1:** The optimized feed forward architecture (neurons distribution) as a function of data set sizes. The data set with the label ${N}_{g}$ has a total of ${N}_{g}^{3}$ data points.

*N*_*g*_	Architecture
3	3 × 3 × 13
4	4 × 4 × 3
5	3 × 7 × 7
6	5 × 6 × 18
7	12 × 8 × 7
8	13 × 11 × 7
9	19 × 20 × 11
10	18 × 18 × 14

**Figure 3:**
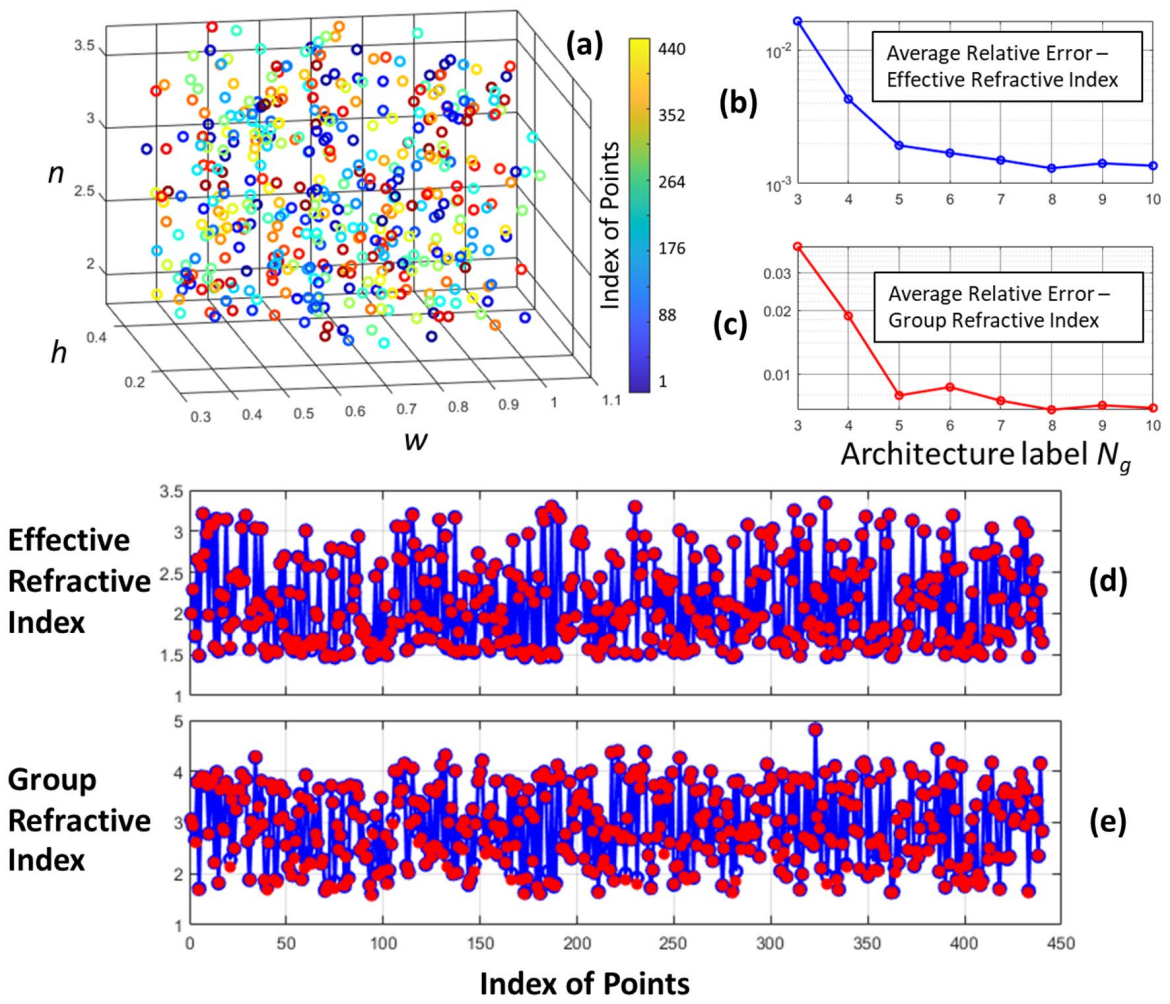
Performance in the general photonic parameter space. (**a**) The location of test data points in the input parameter space. (**b**)Average relative error—effective refractive index, and (**c**) Average relative error—group refractive index for the test data set as a function optimized architecture (neuron distribution). The neuron layout for each *N*_*g*_ can be found from Table 1. (**d**) The exact (blue) versus prediction [with *N*_*g*_ = 8] (red) of effective refractive indices for all the 400 points. (**e**) The exact (blue) versus prediction [with *N*_*g*_ = 8] (red) of group refractive indices for all the 400 points.

For the current work to compute the group refractive index, we independently prepare a set *N* = 400 data points constructed using exact numerical solution to Maxwell’s equation via the method finite differences. We employed the commercially available Lumerical MODE solver and configured a computational window of 5 × 5 µm with metallic boundary conditions^32^. The 400 data points are randomly sampled in the input parameter space (see Fig. 3a). These 400 points cover a wide range of materials, and geometrical parameters in photonics. Figure 3b depicts the average relative error in effective refractive index $\frac{1}{N}{\sum }_{i=1}^{N}\frac{\left|{n}_{eff,predict}-{n}_{eff,exact}\right|}{{n}_{eff,exact}}$ for all the architectures tabulated in Table 1. Figure 3c shows similar metric $\frac{1}{N}{\sum }_{i=1}^{N}\frac{\left|{n}_{g,predict}-{n}_{g,exact}\right|}{{n}_{g,exact}}$ for the group refractive index. Here, the ${n}_{g,predict}$ is computed using Eq. (2) and AD, ${n}_{g,exact}$ is the exact value calculated from the wavelength sweep of numerically evaluated effective refractive indices. The lowest relative error is obtained for the optimized architecture for *N*_*g*_ = 8. This architecture has a neuron distribution of 13 × 11 × 7 (see Table 1). Figure 3d,e visualizes the prediction of the model with *N*_*g*_ = 8 for all the all the 400 data points. The predictions are shown in red colored circles, and the exact numerical evaluations are shown in blue colored circles. As we can see from this figure, the results AD is in very good agreement with respect to the exact evaluations. The training time for the neural network with *N*_*g*_ = 8 is about ~ 20 s. This duration is measured when the training is performed using MATLAB’s *fitnet* function^33^ on a Window workstation with Intel Xenon E5 2.3 GHz processor and 192 Gb of RAM. Further details on the training time for the other values *N*_*g*_ can be obtained from Ref.^29^. For the group refractive index calculation, we developed a separate python program and used AD library from Tensorflow^15^. The neural network weights from the MATLAB model are imported to the python program. On the same workstation, the group index calculation for all the 400 points took a time duration of ~ 0.1 s.

**Figure 4:**
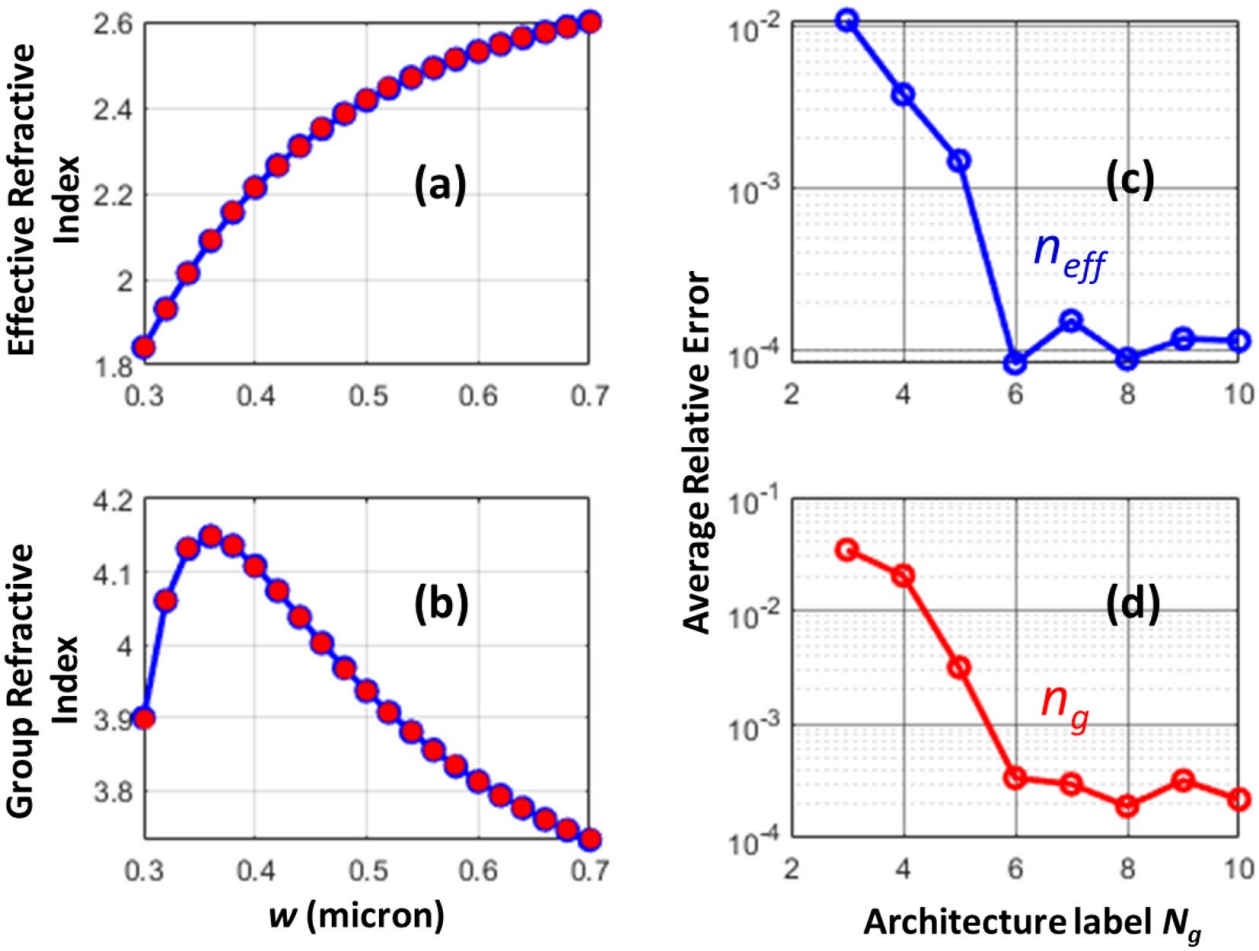
Silicon waveguides (*h* = 0.22 μm) with varying waveguide width (*w*). (**a**) Effective refractive index – exact (blue) versus prediction [*N*_*g*_ = 6]. (**b**) Group refractive index – exact (blue) versus prediction [*N*_*g*_ = 6]. (**c**) Average relative error in effective refractive index predictions for various architectures. (**d**) Average relative error in group refractive index predictions for various architectures.

Figure 4 shows the application of AD for prediction of group refractive indices for typical waveguide dimensions used in silicon photonics. The height of the silicon waveguide is kept at 0.22 µm, and the wavelength of interest is 1.55 micron. The blue color curves in Fig. 4a,b are the exact evaluations of effective and group refractive indices, respectively. The red solid circles are the results of Eq. (2) with model *N*_*g*_ = 6. From Fig. 4c,d we can visually see no discrepancies between the exact and predicted values of the effective and group refractive indices. The average relative errors versus *N*_*g*_ are shown in Fig. 4c,d. From this figure, we see that the architecture corresponding *N*_*g*_ = 6 yields the lower error.

**Figure 5:**
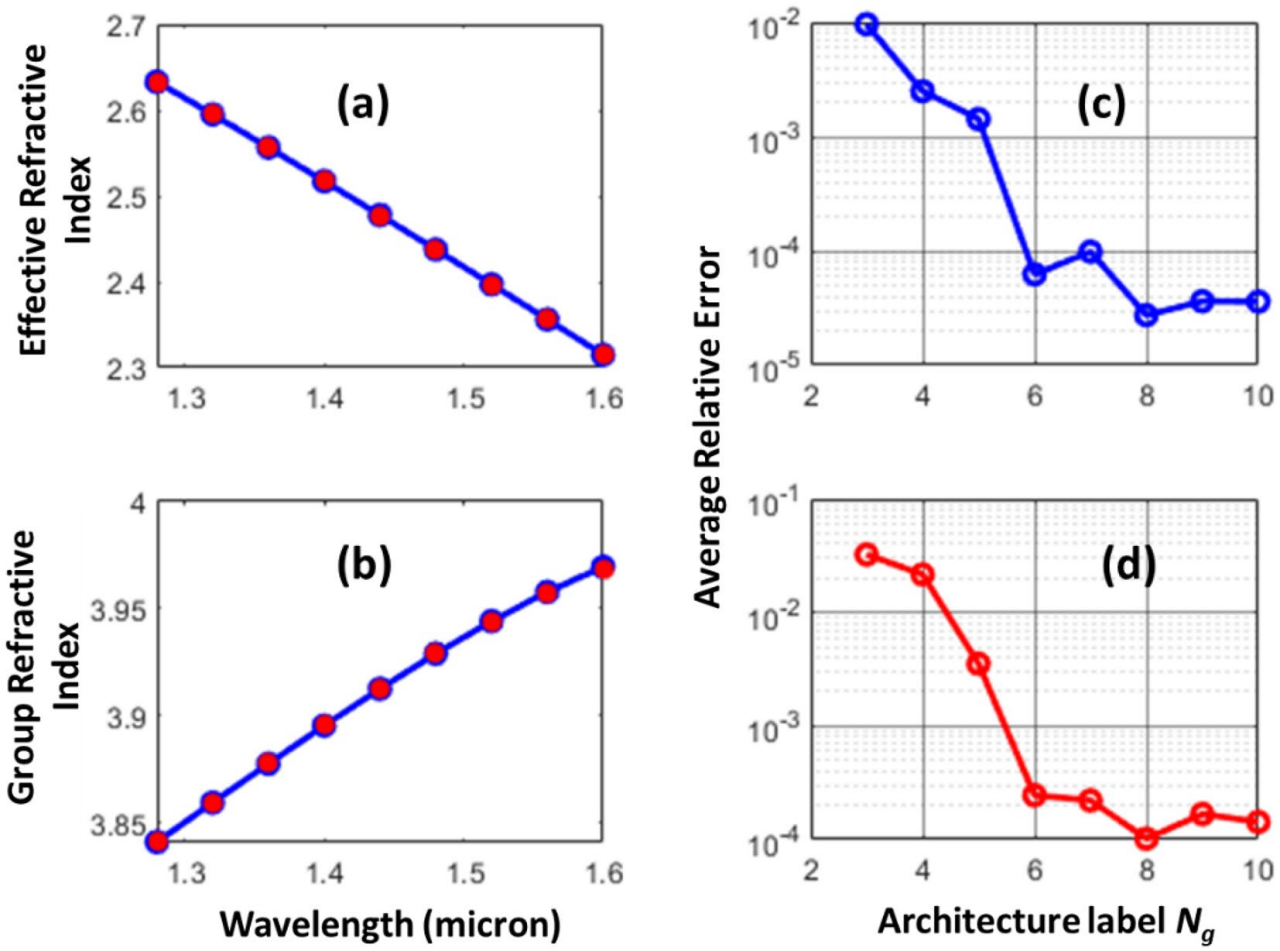
Wavelength response of a standard single silicon mode waveguide with *w* = 0.5 μm, and *h* = 0.22 μm. (**a**) Effective refractive index—exact (blue) versus prediction [*N*_*g*_ = 6]. (**b**) Group refractive index—exact (blue) versus prediction [*N*_*g*_ = 6]. (**c**) Average relative error in effective refractive index predictions for various architectures. (**d**) Average relative error in group refractive index predictions for various architectures.

Figure 5 demonstrates another example in silicon photonics. Here, we calculate the wavelength response of the standard single mode channel waveguide with *w* = 0.5 µm and *h* = 0.22 µm. In Fig. 5a, we show the exact [blue solid line] and the deep learning predicted [red solid circles] effective refractive indices as a function of wavelengths. In the exact calculations, the numerical finite difference simulation has to be repeated for each wavelength. In the deep learning, the same model is used to predict the effective refractive index for all wavelengths, however with a scaled width, $w{\lambda }^{^{\prime}}/\lambda$ and heights, $h{\lambda }^{^{\prime}}/\lambda$. As we can clearly see from Fig. 5a both exact and deep learning evaluations are in good agreement. Figure 5b shows the wavelength response group refractive index. The group refractive indices obtained from ADs via Eq. (2) are shown in red asterisks. Again, these evaluations are in good agreement with exact evaluations [blue solid line]. For the architecture of the deep learning model, we select the architecture with *N*_*g*_ = 6. This architecture has a neuron layout of 5 × 6 × 18 (see Table 1) and yields the lowest error (see Fig. 5c,d).

Neural network uses composition of simple functions and delivers a systematic framework for discovery of a more complicated function that maps the input and output quantities^18,34^. In this context, it is said to be a universal function approximator. This idea is used here to model the relationship between the effective refractive indices and various waveguide parameters. Neural network’s function approximation capability can be exploited to solve a wide range of differential equations. If the differential equation can be casted as $\mathcal{F}\left(u\right)=0$, with $\mathcal{F}$ and $u$ being the differential operator and the function to be solved, respectively, then the solution can be sought by setting the neural network’s loss function as $\mathcal{F}$ and minimizing it via backpropagation algorithm. The differential operations of $u$ in $\mathcal{F}\left(u\right)$ can be calculated using automatic differentiations. The technique of solving differential equations using neural network is first introduced by Lagaris et al.^19,20^ and received a great interest after the term physics informed neural network is coined by Raissi et al.^21–23^. For recent methods and trends in solving differential equations please see Ref.^18^, and the review article^24^. In this article, the relationship between the effective refractive index, wavelength and group refractive index is governed by the differential equation (Eq. 1). However, the task here is not to solve the differential equation and find the corresponding expression of effective refractive index as a function of wavelength, but transforming the equation such that the wavelength derivatives can be evaluated using existing surrogate models.

In summary, we have presented a sophisticated framework for computation of group refractive index of the optical channel waveguides for the entire and usual parameter space of photonics. Deep learning models constructed for effective refractive index predictions are generalized for arbitrary wavelengths by including a wavelength scaling layer, and automatic differentiation is employed to compute the group refractive index without any further training. In a nutshell, the work establishes a new ultrafast methodology for computation of optical modes, effective and group refractive indices. This work demonstrates, for the first time, how derived quantities in optics can be readily evaluated from the parent quantities using automatic differentiation technique and the underlying physics, for standard waveguide geometries and materials in photonics.

## Data Availability

The datasets used and/or analysed during the current study available from the corresponding author on reasonable request.
